# Medical and Dental Students’ Perception of Interdisciplinary Knowledge, Teaching Content, and Interprofessional Status at a German University: A Cross-Sectional Study

**DOI:** 10.3390/ijerph20010428

**Published:** 2022-12-27

**Authors:** Berit Hackenberg, Maximilian-Niclas Schlich, Haralampos Gouveris, Christopher Seifen, Christoph Matthias, Guglielmo Campus, Thomas Gerhard Wolf, Muthuraman Muthuraman, James Deschner

**Affiliations:** 1 Department of Otorhinolaryngology, Head and Neck Surgery, University Medical Center Mainz, 55131 Mainz, Germany; 2 Department of Periodontology and Operative Dentistry, University Medical Center of the Johannes Gutenberg-University Mainz, 55131 Mainz, Germany; 3 Department of Restorative, Preventive and Pediatric Dentistry, School of Dental Medicine, University of Bern, 3010 Bern, Switzerland; 4 Department of Surgery, Microsurgery and Medicine Sciences, School of Dentistry, University of Sassari, 07100 Sassari, Italy; 5 Neural Engineering with Signal Analytics and Artificial Intelligence (NESA-AI), Department of Neurology, University Clinic Würzburg, 97080 Würzburg, Germany

**Keywords:** curriculum, dental students, interprofessional education, knowledge, medical students, perception, teaching

## Abstract

Although oral health is considered a key indicator of overall health, dentistry is still neglected in medical education at the university level. Interprofessional education (IPE) is an important tool to promote collaboration among health care providers and to reduce barriers to access in health care. In this cross-sectional study, medical and dental students at Mainz University, Germany, were surveyed regarding their perception of interdisciplinary knowledge, teaching content, interprofessional standing, and attitudes toward IPE. Spearman’s rank correlation was used to identify associated statements. Structural equation modeling (SEM) was performed to understand how sex, study progress, and prior education might influence student attitudes. In total, 426 medical students and 211 dental students were included in the study. Dental students rated their interdisciplinary knowledge higher than medical students. The relevance of IPE as assessed by the students correlated significantly with their motivation to continue IPE after graduation. Both groups of students valued the other discipline but rejected a combined graduate program. Students with prior professional training valued the synergy of medicine and dentistry more the students without prior training. Interprofessional knowledge and interest in IPE was higher among dental students. Understanding students’ attitudes toward IPE is an important prerequisite for adapting university curricula to strengthen students’ attitudes and motivation.

## 1. Introduction

Oral health, a key indicator for overall health by the WHO [1], is not only essential for overall well-being and a good quality of life, but also associated with systemic diseases [2,3,4]. Despite this, oral health issues are still neglected in health care [5]. Facilitating access to dental care through collaboration between physicians and dentists can improve oral and systemic health, particularly to vulnerable populations [6]. 

Medicine and dentistry are two separate professions even at university level. The merging of courses, especially at the preclinical level, has been under discussion for a long time and could already be implemented at various universities, but the German federal government, for example, rejected a general decision to do so in its last reform of the dental curriculum in 2017 [7]. 

Interprofessional education (IPE) refers to collaborative learning between two or more different healthcare professions [8]. It is considered an effective tool for improving communication and collaboration skills between healthcare providers [9]. Educating all health care providers about oral health issues and promoting interprofessional care can improve health care and reduce the burden of disease [10,11]. Strengthening collaboration between physicians and dentists will reduce barriers to accessing care, improve health outcomes, and reduce treatment costs [12]. Despite these benefits, IPE is still too rarely used. There are few studies on the medical and dental students’ perspectives on IPE [13,14,15]. 

The study period appears to be a sensitive time window to motivate future professionals to pursue IPE. It seems important to ask medical and dental students directly for their opinions, as IPE has a direct impact not only on their daily studies, but also on their future approach to patient care [16]. IPE is a field that is sensitive to different modes of communication, cultures, and different perspectives on traditional hierarchies, boundaries, and responsibilities [17]. We assume that consequences derived from surveys on IPE will be better accepted by students when they come from their direct peers.

This study explores the status of IPE at a German university by surveying medical and dental students about their perceptions of interdisciplinary knowledge, teaching content, interprofessional status of medical and dental professions, and their attitude toward interprofessional education.

## 2. Materials and Methods

This cross-sectional study was conducted at the medical/dental faculty at the Johannes Gutenberg-University of Mainz, Germany, and took place between November 2020 and March 2021. An original standardized questionnaire about student perception in the following areas: interdisciplinary knowledge, teaching content, interprofessional standing, and about their attitude toward interprofessional education was prepared and submitted to medical and dental students. 

### 2.1. Questionnaire and Survey

Questionnaires were distributed to both dental and medical students during lectures. Due to the COVID-19 pandemic, classes were interrupted, and all lectures were switched to an online format. Therefore, the survey was distributed via an online survey tool on various online learning and communication platforms for students of medicine and dentistry at the Johannes Gutenberg-University of Mainz, Germany. 

The questionnaire was divided into five parts (A–E). Part A general information about the students, their academic backgrounds, and their intended specialty; while parts (B–E) were divided into statements about the students’ assessment of interdisciplinary knowledge (B), teaching content (C), perceived interprofessional value and social role of both professions (D), and their view on IPE for dental and medical students (E). All statements had to be rated on a five-point Likert scale between “strongly agree” = 1 and “strongly disagree” = 5. Table 1 shows the items asked in parts A-E for the complete questionnaires. The questionnaires for medical and dental students did not differ in content but were formulated so that the content matched the respective fields of study and that the respective profession had to be valued. 

The survey was anonymized and in German language. Ethical review and approval by the local Institutional Review Board (Ethics Commission of the State Chamber of Physicians of Rhineland-Palatinate) were not required.

### 2.2. Internal Validation and Test–Retest Reliability

For internal validation and test–retest reliability, eight medical and eight dental students were asked to complete the same questionnaire twice, two weeks apart. Responses for each question separately and repeated the Spearman rank correlation matrices for both groups and an Intraclass Correlation Coefficient (ICC) was run to test the stability of our correlation results. An ICC-value of 0.80 or higher was considered satisfactory.

### 2.3. Data Analysis

We calculated Spearman’s rank correlation coefficients between the questionnaires separately for each question between the two groups (medical students and dental students). To accommodate for the high number of correlations, FDR-correction (FDR = false discovery rate) was applied for the comparisons within each modality. The significance threshold was set to *p* < 0.05 (two-tailed).

The structural equation modelling (SEM) analysis was performed in a toolbox for MATLAB (version 13a, Mathworks, Natick, MA, USA). SEM represents a complex analytical tool that enables a determination between the causal relationships of the variables in a model-based approach. Initially, we used a brute force method to model all possible combinations for the input and mediator between the three variables, namely, sex, semester, and pre-education. The relationship between these three variables and the output of questionnaire parts B–E were modelled for each question separately. 

The Maximum Likelihood method of estimation to fit the models was employed. The Root Mean Square Error of Approximation index, which improves precision without increasing bias was used to adjust the models for a large sample size. The index estimates lack of fit in a model compared with a perfect model and therefore should be low. In all models, the Invariant under a Constant Scaling and its factor criteria should be close to zero, which will signify that models were appropriate for analysis. Finally, using the Akaike Information Criterion (AIC), the quality of each model relative to other models was estimated, with smaller values signifying a better fit for the model. The obtained criterion comparing the models varied between 0.02 and 0.04 (which indicates a good fit for the models). The strength of associations between the variables in the models was quantified by standardized coefficients (s), ranging from 0 (no association) to 1 (very strong association). The index for all models was below 0.05, which indicates a very good fit for the models. In addition to the AIC for the multiple models, we have controlled the results, the adjusted Bonferroni correction severity of the adjustment was weakened with an increasing value of the average absolute correlation between two parameters in the model [18]. The described significant models survived the adjusted Bonferroni correction with (*p* < 0.05).

## 3. Results

In total, 436 medical students and 211 dental students participated in the study (of a total of 2930 medical and 540 dental students at the Johannes Gutenberg-University of Mainz, Germany). From group 1 (medical students), 10 participants had to be retrospectively excluded because they had not completed questionnaire part B–E. There were no missing data in these sections for group 2 (dental students).

Medical and dental students did not differ significantly in their mean age of 25.0 years for medical students and 23.8 years for dental students. More participants in both groups were female. In the medical student group, significantly more participants had completed a professional training before entering graduate school. This training was most often in the medical field (e.g., nurse, paramedic). Descriptive statistics of the two groups are shown in Table 2. 

In section B, dental students rated their interdisciplinary knowledge better than medical students (B1–B4, see Table 3). Medical students were more likely to disagree with having good interdisciplinary knowledge (B1–B3). Furthermore, in section C, dental students felt that medical studies were better covered in their courses (C1) and that the two subjects were better connected (C3). They were also more interested in the other subject (C2) and valued interprofessional knowledge higher than their medical peers (C5). Satisfaction with interprofessional collaboration at the university (C3) and their interest medicine and joint courses (C2, C4) did not correlate.

Both disciplines were valued in their interprofessional standing (D1, D2), although medicine was rated higher by both medical and dental students. Dental students appreciated interprofessional knowledge for their future careers more than medical students (D4). In significant correlation, they were also more motivated to continue interprofessional education after graduation (D5). Both medical and dental students rejected a combined graduate program or the option of dentistry being a residency program after medical school (E1, E2). 

Statements as listed in Table 1 were rated on a five-point Likert scale ranging from 1 = strongly agree to 5 = strongly disagree. A lower mean score means a stronger agreement with the given statement. Group 1: medical students, Group 2: dental students, SD = standard deviation.

**Figure 1:**
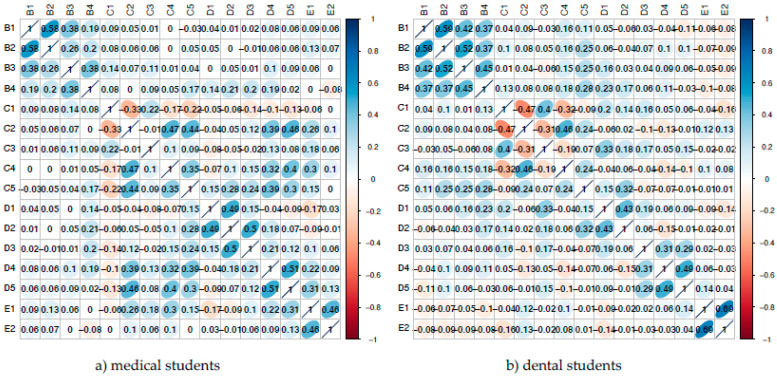
Spearman rank correlation matrices for medical students (**a**) and dental students (**b**) separately for each question in the questionnaire. The color bar indicates the correlation coefficient the dark blue positive correlation and orange colors negative correlation.

In both groups, the strongest correlations were found between single items within the same section (Figure 1). The following statistically significant correlations for both student groups were deemed to be most important in terms of content: relevance of interdisciplinary knowledge (D4) correlating with interdisciplinary education after graduation (D5) as well as clinical interdisciplinary knowledge (B3) correlating with synergy of medicine and dentistry (B4). For medical students, the correlation of own dental knowledge (C2) with importance of dental knowledge in the medical profession (C5) was also significant and of interest in terms of content. For dental students, this was true for the correlations of knowledge in physiology (B2) with synergy of medicine and dentistry (B4) and of relevance of human medicine (D3) with relevance of interdisciplinary knowledge (D4). 

Intersection correlation was strongest for medical students, with relevance of dental knowledge (D4) and own dental education after graduation (D5) correlating significantly with items own dental knowledge (C2) and interprofessional courses (C4). On the other hand, dental students showed the strongest intersection correlation for item importance of medical knowledge in the dental profession (C5), which correlated significantly with the items knowledge in medical physiology (B2), clinical medical knowledge (B3), synergy of medicine and dentistry (B4) and standing of physicians (D2). 

**Figure 2:**
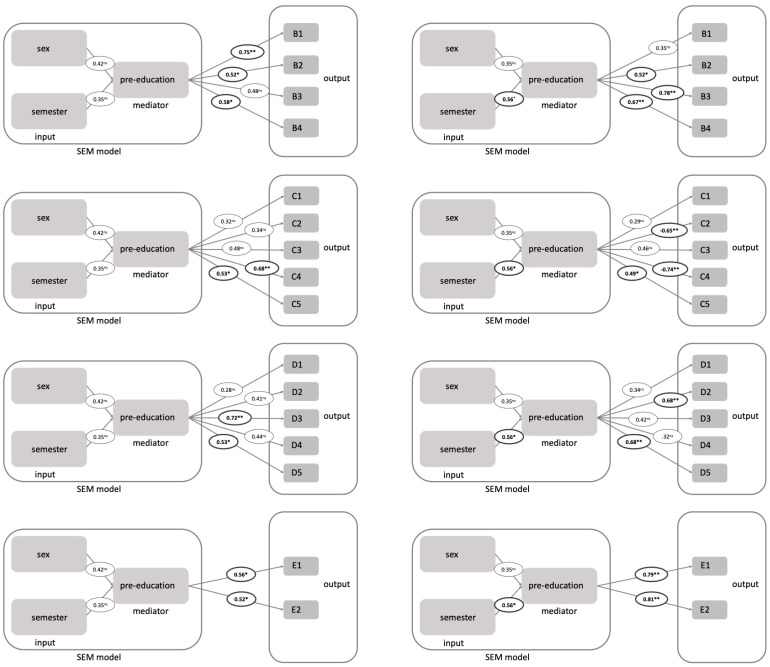
Structural equation modelling for sex, semester as input and pre-education as mediator and the corresponding prediction coefficients are written in circles for each question separately. The ** indicates significance *p* < 0.01 and * indicates significance of *p* < 0.05. ns indicates not significant.

Structural equation modelling (SEM) for medical students showed that gender, prior education, and study progress in combination were significant predictive factors for items knowledge of dental anatomy (B1), knowledge in dental physiology (B2), synergy of medicine and dentistry (B4), interprofessional courses (C4), importance of dental knowledge in the medical profession (C5), relevance of dentistry (D3), own dental education after graduation (D5), combined degree program (E1) and dentistry as medical residency program (E2) within the medical student group (Figure 2). When all three input factors were tested separately for the items B4, C4 and D3, only prior education was a significant predictor for all of these items whereas the semester group predicted only item synergy of medicine and dentistry (B4). Medical students with prior education tended to agree more with items synergy of medicine and dentistry (B4), interprofessional courses (C4) and relevance of dentistry (D3) than students without prior education (mean values for previous education yes/no for B4: 2.28/2.49, C4: 3.21/3.25, D3: 1.92/2.04). Item B4 found more agreement with increasing semester group (mean values for semester group 1/2/3: 2.47/2.35/2.32). 

For dental students, these three factors in combination (gender, prior education, semester group) were significantly predictive for items knowledge in medical physiology (B2), clinical medical knowledge (B3), synergy of medicine and dentistry (B4), own medical knowledge (C2), interprofessional courses (C4), importance of medical knowledge in the dental profession (C5), standing of physicians (D2), own medical education after graduation (D5), combined degree program (E1) and dentistry as medical residency program (E2) (Figure 2). For items B3, B4 and C4, both semester group and prior education, tested separately, were significantly predictive. Gender was, on the other hand, not a predictive factor. Dental students with prior education tended to agree more with items B3, B4 and C4 (mean values for previous education yes/no for B3: 1.81/2.33, B4: 1.75/1.90, C4: 3.08/3.11). For items B3 and B4, agreement increased with increasing study progress (mean values for semester group 1/2/3 for B3: 2.51/2.27/1.84, B4: 1.98/1.94/1.67). For item C4, this was reversed (mean values for semester group 1/2/3 for C4: 2.98/3.26/3.03).

**Figure 3:**
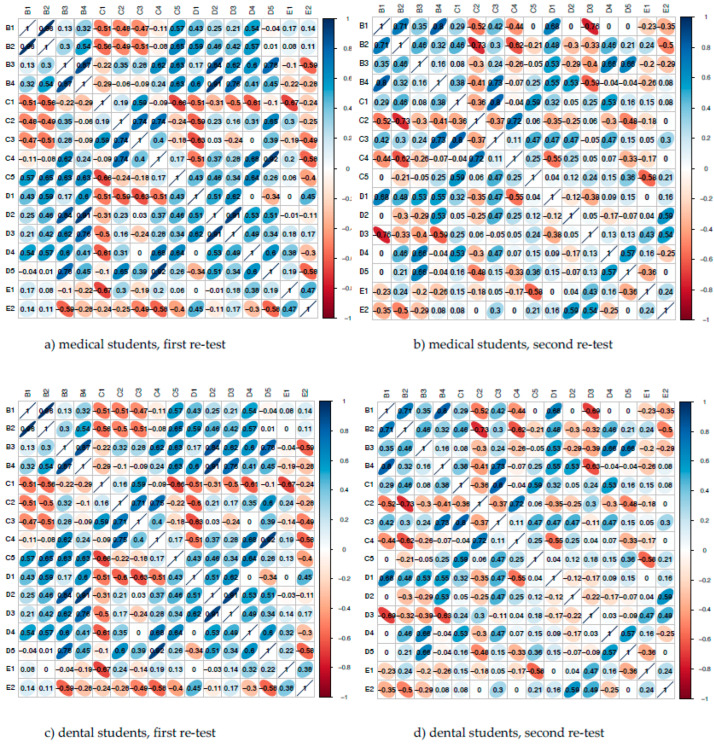
Spearman rank correlation matrices for medical students and dental students surveyed for internal questionnaire validation (**a**) medical, and (**c**) dental and again after a two-week interval (**b**) medical, and (**d**) dental. The color bar indicates the correlation coefficient: dark blue, positive correlation; orange, negative correlation.

Re-application of both questionnaires twice two weeks apart showed good test–retest reliability of the results. All responses except two within the medical student group were the same. The exceptions were: item C2 (2 vs. 3) and item E1 (5 vs. 4). Spearman rank correlation matrices for both groups showed stable results to the above-described correlation results (see Figure 3). 

## 4. Discussion

In this survey, dental students rated their interdisciplinary knowledge higher than medical students. They also rated the relevance of IPE higher, which correlated significantly with their motivation to continue IPE after graduation. 

Interprofessional knowledge is an important tool for improving healthcare overall. The merging of medical and dental degree programs has often been discussed but has so far been rejected in most countries [19,20,21,22]. A better understanding of the current status of IPE in universities will help us set the course for improving its place in future education. In this study, we surveyed medical and dental students across different years of study about their interdisciplinary knowledge, teaching content, interprofessional standing and attitudes toward interprofessional education. 

To best of the authors’ knowledge, this study is the first to use Spearman’s rank correlation and SEM to understand the complex relationships in this area of study. This multifaceted evaluation was made possible by the large cohort size. Furthermore, this study included students from different semester groups to reflect and present comprehensive opinions during study. 

The high rate of interdisciplinary knowledge among dental students was previously described [21], where dental students rated their medical knowledge as moderate to good, while medical students rated their dental knowledge as poor to moderate [23]. A study from the United States of America that surveyed 316 dental and 187 medical students also found that dental students had higher knowledge of oral health and interprofessional collaboration and had more positive attitudes toward IPE [24]. In addition, dental students felt that medical studies were better covered in their courses and that the two subjects were better connected. This difference between dental and medical students could be because the dental curriculum includes several medical courses, whereas the medical curriculum does not cover dental issues [25]. 

Furthermore, dental students valued IPE more highly than medical students for their future careers. Among medical students, the rating of the relevance of dental knowledge correlated with the desire for more interprofessional study courses. This results in the need for more dental courses in medical school. It appears to be particularly important given that medical students view their education as fair or poor in preparing them for oral health-related patient care [26,27]. 

The correlation model shows that both medical and dental students who have basic knowledge of common dental/medical diseases also understand better how medicine and dentistry intersect. Therefore, dental/medical knowledge should be part in both curricula to create a better understanding of how the two disciplines interact. This understanding is a prerequisite for IPE. In addition, students who perceive the other discipline as relevant to their future professional life are also motivated to continue IPE after graduation. 

Medical students who agreed that physicians should have basic dental knowledge also desired more knowledge in the field of dentistry. Thus, valuing dentistry as an integral part of overall health appears to be an important motivating factor for IPE. Dental students who had a basic knowledge of common medical diseases also felt they had a better understanding of how these two disciplines intersect in some diseases. In addition, those who viewed human medicine as relevant to oral health also valued knowledge in this area as relevant for their future careers. Teaching interprofessional knowledge at the university level seems to be an important way to build the foundation of interest in IPE that could accompany the students during their future work as physicians and dentists. 

Medical and dental students with prior education before entering university were more likely to agree that they have knowledge of basic medical/dental diseases and understand how these two subjects intersect. In addition, medical students with prior education rated dentistry as more relevant to overall health, and dental students with prior education desired for more courses together. Students with prior education might rate IPE higher because they often have prior professional experience and may have witnessed the benefits of interprofessional practice firsthand. Our finding is consistent with previous literature and supports the fact that previous education improves the chances of getting into a university [13,28]. Both groups of students had an increased understanding of the interplay between medicine and dentistry as their studies progressed. Gender, on the other hand, was not a significant predictor in SEM. In contrast, two Swedish studies found that female students were significantly more positive about IPE than male students [29,30]. Both studies included medical but not dental students and therefore provide only a one-sided view. 

The main strength of our study is its large cohort size, which allows a detailed statistical analysis. When looking at the distribution of the two groups studied, the percentage distribution in relation to the total number of all students of the respective field of study at the University of Mainz is significantly higher for dental students compared with medical students. The reason for the uneven distribution could be that the pandemic-related interruption and distribution of questionnaires during lectures was no longer carried out, as face-to-face classes were banned by the state and switched to online formats. However, the usage behavior of each format was not verified, but could have resulted in the motivation to participate through the numerous online formats using online learning and communication platforms being less popular with students and therefore less used. Unfortunately, a central distribution by the authority of the university was not possible due to data protection regulation. Furthermore, by including students of different semesters, we were also able to show a development over the course of studies. However, several limitations of this study should be mentioned. First, the questionnaire used was created by the authors because there was no validated German questionnaire in the literature. Furthermore, we conducted the study at the Johannes Gutenberg-University in Mainz, Germany. As a public university, it is one of the largest universities in Germany [31]. Due to the classical structure of study programs in medicine and dentistry, we consider the University of Mainz to be representative for the study situation in Germany. Nevertheless, the results reflect the opinion of students at this one German university. However, we would like to point out the fact that human medicine and dentistry are separated at university level in many countries especially in the clinical area from the 3rd year of study onwards. Future studies are needed to evaluate IPE to different phases of the study as well as postgraduate. 

## 5. Conclusions

The relevance of IPE as rated by students correlated with their motivation to continue IPE after graduation. Dental students rated their interdisciplinary knowledge higher than medical students. Medical and dental students valued the other discipline but rejected a combined graduate program. Students with prior professional training valued the synergy of medicine and dentistry more than students without prior training. Understanding students’ attitudes toward IPE is an important prerequisite for adapting university curricula to this goal. We believe that academic education is a critical time to motivate and inform students, as well as to advocate for a more interdisciplinary curriculum.

## Figures and Tables

**Table 1:** Items of both questionnaires for medical and dental students.

Item	Statement	Version	
		1	2
B1	“I have good knowledge of the anatomy of the oral cavity & teeth.”	✓	
	“I have good knowledge of the human anatomy.”		✓
B2	“I have good knowledge of the functioning of the jaw and masticatory muscles.”	✓	
	“I have good knowledge of the functioning of the heart and circulation.”		✓
B3	“I have basic knowledge of common dental diseases (caries, periodontitis).”	✓	
	“I have basic knowledge of common medical diseases (hypertension, diabetes).”		✓
B4	“I understand how human medicine & dentistry overlap in some diseases.”	✓	✓
C1	“Dentistry is adequately addressed in the study of human medicine.”	✓	
	“Human medicine is adequately addressed in the study of dentistry.”		✓
C2	“I would like to be more knowledgeable about dentistry.”	✓	
	“I would like to be more knowledgeable about human medicine.”		✓
C3	“Dentistry and human medicine are structurally linked at our university.”	✓	✓
C4	“I would like to see more courses with dentistry students together.”	✓	
	“I would like to see more courses with human medicine students together.”		✓
C5	“Human physicians should have basic dental knowledge.”	✓	
	“Dentists should have basic medical knowledge.”		✓
D1	“Human physicians have a leading role in medicine.”	✓	
	“Dentists have a leading role in medicine.”		✓
D2	“Dentists have a leading role in our society.”	✓	
	“Human physicians have a leading role in our society.”		✓
D3	“Dentistry is just as relevant to the overall health as human medicine is.”	✓	
	“Human medicine is just as relevant to the oral health as dentistry is.”		✓
D4	“Dental knowledge is highly relevant for my future professional life”.	✓	
	“Human medicine knowledge is highly relevant for my future professional life”.		✓
D5	“I would like to continue my education in dentistry even after I graduate.”	✓	
	“I would like to continue my education in human medicine even after I graduate.”		✓
E1	“Dental and human medicine should be structured as a unified degree program.”	✓	✓
E2	“Dentistry should be offered as a residency after human medical school.”	✓	✓

The statements listed above were rated by students based on their level of agreement on a five-point Likert scale (1 = strongly agree to 5 = strongly disagree). Items are numbered B1–E2 depending on the section they appeared in. Group 1: medical students, Group 2: dental students. ✓: version used for medical and/or dental students.

**Table 2:** Descriptive statistics.

	**Group 1: Medical Students**	**Group 2: Dental Students**
*n*	426	211
Age	25.0 (3.8)	23.8 (3.2)
Gender, *n* (%) male	147 (34.5)	64 (30.3)
Previous education, *n* (%) yes	232 (54.5) *	55 (26.1) *
Semester group (1–3), mean	1.97	2.05

* *p* < 0.05, SD = standard deviation; previous education = completed training (e.g., nurse, paramedic, dental assistant) before medical or dental school; semester group/medical students = 1 (semester 1–4), 2 (semester 5–8), 3 semester 9–12); semester group/dental students = 1 (semester 1–3), 2 (semester 5–7), 3 (semester 9–10).

**Table 3:** Mean results (standard deviation) of questionnaire part B–E for both groups.

Item	Group	Mean (SD)	Item	Group	Mean (SD)
B1	1	3.4 (1.0) *	D1	1	1.4 (0.8) *
	2	2.2 (0.7) *		2	1.9 (0.9) *
B2	1	3.1 (1.1) *	D2	1	1.7 (0.9) *
	2	2.2 (0.8) *		2	1.4 (0.8) *
B3	1	3.2 (1.2) *	D3	1	2.0 (1.1) *
	2	2.2 (0.8) *		2	2.2 (1.0) *
B4	1	2.4 (1.1) *	D4	1	3.4 (1.0) *
	2	1.9 (0.8) *		2	2.0 (0.9) *
C1	1	3.5 (1.1) *	D5	1	4.0 (1.0) *
	2	2.4 (1.0) *		2	2.7 (1.3) *
C2	1	3.2 (1.2) *	E1	1	4.0 (1.1) *
	2	2.5 (1.1) *		2	3.3 (1.3) *
C3	1	3.4 (1.0) *	E2	1	3.4 (1.3) *
	2	2.5 (0.8) *		2	3.7 (1.3) *
C4	1	3.2 (1.0)			
	2	3.1 (1.1)			
C5	1	2.2 (1.1) *			
	2	1.5 (0.7) *			

* *p* < 0.05, difference considered statistically significant, two-sample t-test assuming unequal variances.

## Data Availability

Not applicable.
